# Comparison of the predictive performance of risk of malignancy indexes 1–4, HE4 and risk of malignancy algorithm in the triage of adnexal masses

**DOI:** 10.1186/s13048-020-00643-6

**Published:** 2020-04-25

**Authors:** Abha Hada, Li-ping Han, Yanyan Chen, Qing-hong Hu, Yidan Yuan, Liya Liu

**Affiliations:** Department of Obstetrics and Gynecology, The First Affiliated Hospital of Zhengzhou University, Zhengzhou, 450002 P. R. China

**Keywords:** Adnexal mass, Risk of malignancy index, Risk of malignancy algorithm, Human epididymis protein 4, Ovarian cancer

## Abstract

**Objectives:**

For patients presenting with adnexal mass, it is important to correctly distinguish whether the mass is benign or malignant for the purpose of precise and timely referral and implication of correct line of management. The objective of this study was to evaluate the performance of Risk of malignancy Indexes (RMI) 1–4, Human Epididymis Protein 4 (HE4) and Risk of Malignancy Algorithm (ROMA) in differentiating the adnexal mass into benign and malignant.

**Methods:**

A retrospective study using 155 patients diagnosed with adnexal mass between January 2014 to December 2014 in The First Affiliated Hospital of Zhengzhou University was conducted. The patient records were assessed for age, menopausal status, serum CA125 and HE4 levels, ultrasound characteristics of the pelvic mass and the final pathological diagnosis of the mass. RMI1, RMI2, RMI3, RMI4, ROMA were calculated for each patient and the sensitivity, specificity and the Receiver Operating Characteristics (ROC) curves were determined for each test to evaluate their performance.

**Results:**

Among 155 patients with adnexal masses meeting inclusion criteria, 120 (77.4%) were benign, 8 (5.2%) borderline and 27 (17.4%) were malignant. RMI2 and RMI4 had the highest sensitivity (66.7%) while HE4 had the highest specificity (96.9%).Although ROMA had the highest area under the curve (AUC) of 0.886 it was not found to be statistically superior to the other tests. For epithelial ovarian cancers, ROMA (80%), HE4 (96.9%) and RMI 4 (0.868) had the highest sensitivity, specificity and AUC respectively however, the AUC characteristics were not statistically significant between any groups. Compared to the postmenopausal group (sensitivity 72.2–77.8%) all the tests showed lower sensitivity (42.9%) for the premenopausal group of patients.

**Conclusions:**

RMI 1–4, ROMA and HE4 were all found to be useful for differentiating benign/borderline adnexal masses from malignant ones for deciding optimal therapy, however no test was found to be significantly better than the other. None were able to differentiate between benign and borderline tumors. All of the tests demonstrated increased sensitivity when borderline tumors were considered low-risk, and when only epithelial ovarian cancers were considered.

## Introduction

Ovarian cancer is the seventh most common cancer worldwide in females and the 18th most common cancer overall [1]. Its presentation with non-specific symptoms and lack of screening strategies result in delay in diagnosis which is frequently made in advanced stage, has made it a great clinical challenge among all gynecological cancers till now with the overall 5-year survival of 44% (92% for Stage I versus 27% for Stage IV) [2]. It is crucial to differentiate between benign and malignant pelvic masses so that early and correct referral and optimal treatment can be provided, the effect of which is great on the prognosis. Furthermore, correct identification of benign the masses lessens the burden of inappropriate referral to the tertiary care centers that should focus their efforts on patients with malignancies.

Various efforts have been made to develop a system that will help to accurately differentiate a pelvic mass as benign or malignant. In 1990, Jacobs et al. developed the Risk of Malignancy Index (RMI), a risk scoring system based on menopausal status, CA125 levels and ultrasound characteristics with a sensitivity of 85.4% and a specificity of 96.9% when using a cut-off level of 200 to indicate malignancy [3]. Tingulstad et al. modified the original RMI and developed RMI2 in 1996 [4]. In 1999 the RMI 3 was developed with further modification in the scoring of ultrasound score (U) and menopausal status (M) [5]. In 2009, Yamamoto et al. developed the RMI 4 which included tumor size (S) in the RMI [6].

Human epididymis protein 4 (HE4) is a glycoprotein first identified in the epithelium of the distal epididymis belonging to the family of whey acidic four-disulfide core (WFDC) proteins. HE4 is expressed in low amount in normal tissues including epithelia of respiratory and reproductive tissues, [7] overexpressed in > 90% of serous and endometrioid epithelial ovarian cancers and 50% of clear cell carcinomas but not in mucinous ovarian carcinomas [8]. Although its level may be increased in other malignancies such as lungs, colon and breast, highest levels are found in ovarian cancers. Moore et al. developed the Risk of Ovarian Malignancy Algorithm (ROMA), combining two biomarkers: HE4 and CA125 to categorize the patients into low risk or high risk based on their menopausal status [9].

## Patients and methods

The records of all patients presenting with pelvic mass and managed in The First Affiliated Hospital of Zhengzhou University between January 2014 and December 2014 were identified. In addition to patient demographics, pre-operative CA125 and HE4 levels were determined and the characterization of the mass was done by trans-abdominal and trans-vaginal ultrasonography. Patients were considered postmenopausal if they had at least 1 year of amenorrhea or for those who had undergone hysterectomy, if they were 50 years of age or older. Patients who had history of bilateral oophorectomy, ovarian cancer or any active cancer were excluded from the study. All patients underwent laparoscopic removal of the ovarian mass was and further management was decided based upon either intra operative frozen section results or final pathologic diagnosis. The final outcome was determined based on the histopathological results. Borderline tumors were categorized into the benign group for analysis purposes.

CA125 and HE4 levels were determined on cobas e 601 analyzer from Roche Diagnostics using electrochemiluminescence immunoassay (ELICA). For HE4, a cut off value of 70 pmol/l was used.

RMI calculations were made in the following manner:

RMI calculations were made using menopausal status (M), ultrasound score (U), serum CA125 value and in case of RMI 4 an additional parameter of single greatest diameter of tumor size (cm.) (S) was included. For calculating ultrasound score, each ultrasonographic characteristics of pelvic mass: Multilocularity, solid areas, bilaterality, ascites and intra-abdominal metastases scored one point each and were summed up.

RMI 1 = M*U*CA125 where, M = 1 for (premenopausal)

M = 3 for postmenopausal.

U = 0 for ultrasound score of 0.

U = 1 for ultrasound score of 1.

U = 3 for ultrasound score of > 1

RMI 2 = M*U*CA125 where, M = 1 for (premenopausal)

M = 4 for postmenopausal.

U = 1 for ultrasound score of 0 or 1.

U = 4 for ultrasound score of > 1

RMI 3 = M*U*CA125 where, M = 1 for (premenopausal)

M = 3 for postmenopausal.

U = 1 for ultrasound score of 0 or 1.

U = 3 for ultrasound score of > 1

RMI 4 = M*U*CA125*S where, M = 1 for (premenopausal)

M = 4 for postmenopausal.

U = 1 for ultrasound score of 0 or 1.

U = 4 for ultrasound score of > 1.

S = 1 for single greatest diameter of tumor size < 7 cm.

S = 2 for single greatest diameter of tumor size ≥7 cm.

Cut off of 200 was used for RMI 1, 2 and 3 and that of 450 was used for RMI 4.

ROMA calculation:

The algorithm was calculated using the HE4 and CA 125 values based on the menopausal status of the patient to stratify women into risk groups. A Predictive Index (PI) was calculated for premenopausal and postmenopausal patients separately using equations (1) and (2) respectively. Using Roche Elecsys specificity of 75%, premenopausal women with a ROMA value ≥11.4, had a higher risk of ovarian cancer. Postmenopausal women with ROMA value ≥29.9 had a higher risk of ovarian cancer.

(1) Premenopausal:

$$ \mathrm{PI}=-12.0+2.38\ast \mathrm{LN}\ \left[\mathrm{HE}4\right]+0.0626\ast \mathrm{LN}\ \left[\mathrm{CA}125\right] $$

(2) Postmenopausal:

PI = -8.09 + 1.04*LN [HE4] + 0.732*LN [CA125] where, LN = Natural (Logarithm)

ROMA was calculated using the PI using following equation to calculate the predictive probability:

$$ \mathrm{ROMA}\ \mathrm{value}\ \left(\%\right)=\exp\ \left(\mathrm{PI}\right)/\left[1+\exp\ \left(\mathrm{PI}\right)\right]\ast 100\ \mathrm{where},\exp\ \left(\mathrm{PI}\right)=\mathrm{ePI} $$

Patients were classified as high risk or low risk for epithelial ovarian cancer based on following criteria.

Premenopausal women:

ROMA value ≥11.4% = high risk of finding epithelial ovarian cancer.

ROMA value < 11.4% = low risk of finding epithelial ovarian cancer.

Postmenopausal women:

ROMA value ≥29.9% = high risk of finding epithelial ovarian cancer.

ROMA value < 29.9% = low risk of finding epithelial ovarian cancer.

Statistical analysis was performed using SPSS version 19. The median age of the patients was compared using Mann Whitney test, and categorical variables were compared with the Chi-square (χ2) test. The Mann Whitney test was used to compare the medians of the test values in different groups. Receiver Operator characteristics (ROC) curves were constructed and the areas under the curve (AUC) for each model was compared for their accuracy. The sensitivity, specificity, positive and negative predictive values were calculated for each models using the recommended cut-off values. For all statistical comparisons, a level of *P* < 0.05 was accepted as being statistically significant.

## Result

Out of 155 patients who presented with pelvic mass, 113 (72.9%) were premenopausal and 42 (27.1%) were postmenopausal. The median age of presentation was 41 years (range 14–78). The median age in premenopausal and postmenopausal women was 32 and 53 years respectively. Among them, 120 (77.4%) had benign, 8 (5.2%) borderline and 27 (17.4%) had malignant tumors. The median age of patients with benign, borderline and malignant tumors were 38, 37.5, 52 years respectively. The percentage of benign, borderline and malignant cases in premenopausal women were 82.5, 75 and 29.6% respectively. The percentage of benign, borderline and malignant cases in postmenopausal women were 17.5, 25 and 70.4 respectively showing that malignancy was more common in postmenopausal group (*P*-value< 0.001) (Table 1).

**Table 1:** Baseline characteristics of the sample under study

		Benign	Borderline	Malignant
Number	n(%)	120 (77.4)	8 (5.2)	27 (17.4)
Age (years)	Median (range)	38 (14–78)	37.5 (24–61)	52 (23–72)
Postmenopausal status	n(%)	21 (17.5)	2 (25)	19 (70.4)
RMI1	Median (range)	18.03 (0–1679)	23.06 (0–156)	340.80 (0–19,224)
RMI2	Median (range)	35.94 (4.15–1679)	57.48 (11.07–277.92)	454.4 (12.67–34,176)
RMI3	Median (range)	31.65 (4.15–1679)	48.49 (11.07–158.4)	340.8 (12.67–19,224)
RMI4	Median (range)	52.02 (4.15–3358)	77.80 (11.07–555.84)	908.80 (12.67–68,352)
ROMA	Median (range)	5.9 (1.6–28.5)	8.2 (3.2–60.9)	34.6 (4–99.1)
HE4 pmol/l	Median (range)	42.17 (25.37–123)	47.03 (34.47–163.30)	57.59 (35.57- > 1500)

*RMI* Risk of malignancy index, *ROMA* Risk Of Malignancy Algorithm, *HE4* Human Epididymis Protein 4

The respective median values of RMI1, RMI2, RMI3, RMI4, ROMA and HE4 were significantly different between benign and malignant group (*P*-value< 0.001). No significant difference was seen between the median values between benign and borderline group. Between the median values for borderline and malignant masses, significant difference was observed for all tests except HE4 (Table 2).

**Table 2:** Comparison of medians of the test values between benign, borderline and malignant groups

	RMI 1	RMI 2	RMI3	RMI4	ROMA	HE4
Benign vs malignant *(P*-value)	< 0.001	< 0.001	< 0.001	< 0.001	< 0.001	< 0.001
Benign vs borderline (*P-* value)	0.801	0.355	0.485	0.414	0.209	0.127
Borderline vs malignant (*P-* value)	0.002	0.005	0.005	0.001	0.013	0.166

*RMI* Risk of malignancy index, *ROMA* Risk Of Malignancy Algorithm, *HE4* Human Epididymis Protein 4

Among benign masses, mature teratoma was the most common (31.6% of benign tumors) followed by functional ovarian cysts which includes corpus luteum cysts and follicular cysts and endometrioma. Among borderline tumors, borderline serous tumors was the most common (*n* = 5, 3.5%). Among malignant tumors, epithelial ovarian cancers were the most common (*n* = 15, 9.6, 56% of malignant tumors) and among them, serous cystadenocarcinomas (*n* = 10) were the most frequently observed histological subgroup. Most malignancies were stage II and above. The non-epithelial ovarian cancers were less common and consisted of 5 granulosa cell tumors, 3 sarcomatous tumors, 2 immature teratomas, 1 dysgerminoma and 1 ovarian yolk sac tumors. (Table 3).

**Table 3:** Frequency distribution of the histopathological classification of adnexal mass

Subtype	N	%
Malignant tumors		
Serous cystadenocarcinoma	10	6.5
Mucinous cystadenocarcinoma	2	1.3
Endometroid adenocarcinoma	1	1.9
Sarcomatous tumor	3	0.6
Immature teratoma	2	1.3
Granulosa cell tumor (malignant)	5	3.2
Dysgerminoma	1	0.6
Ovarian yolk sac tumor	1	0.6
Fallopian tube adenocarcinoma	1	0.6
Peritoneal carcinoma	1	0.6
Benign tumors		
Mature teratoma	38	24.5
Benign ovarian cysts	32	20.6
Endometrioma	25	16.1
Endometriod tumor	6	3.9
Serous cystadenoma	7	4.5
Mucinous cystadenoma	6	3.9
Tubo-ovarian abscess	5	3.2
Leiomyoma	1	0.6
Borderline tumors		
Borderline serous cystadenoma	5	3.2
Borderline mucinous cystadenoma	3	1.9
Total	155	100

**Fig. 1:**
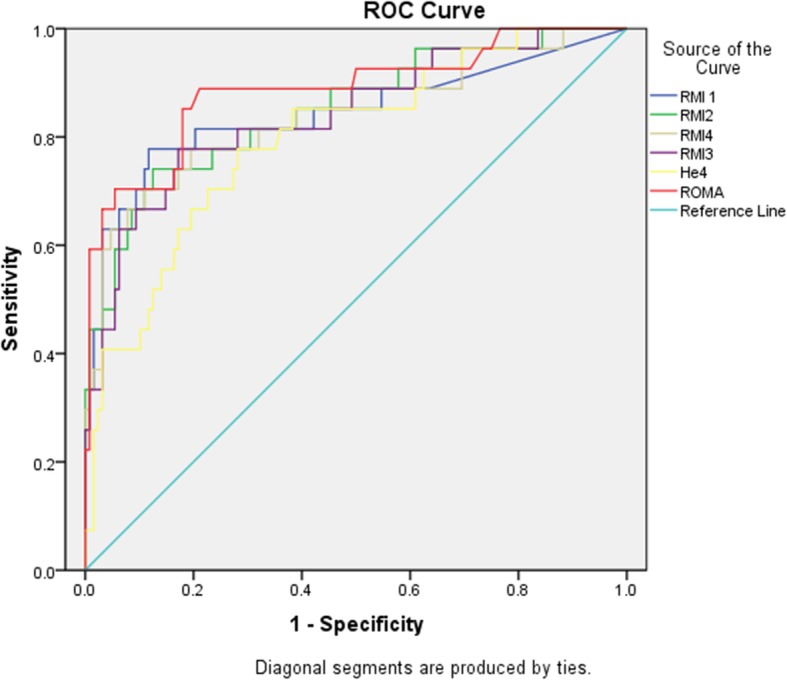
ROC curve benign vs all ovarian cancers

**Fig. 2:**
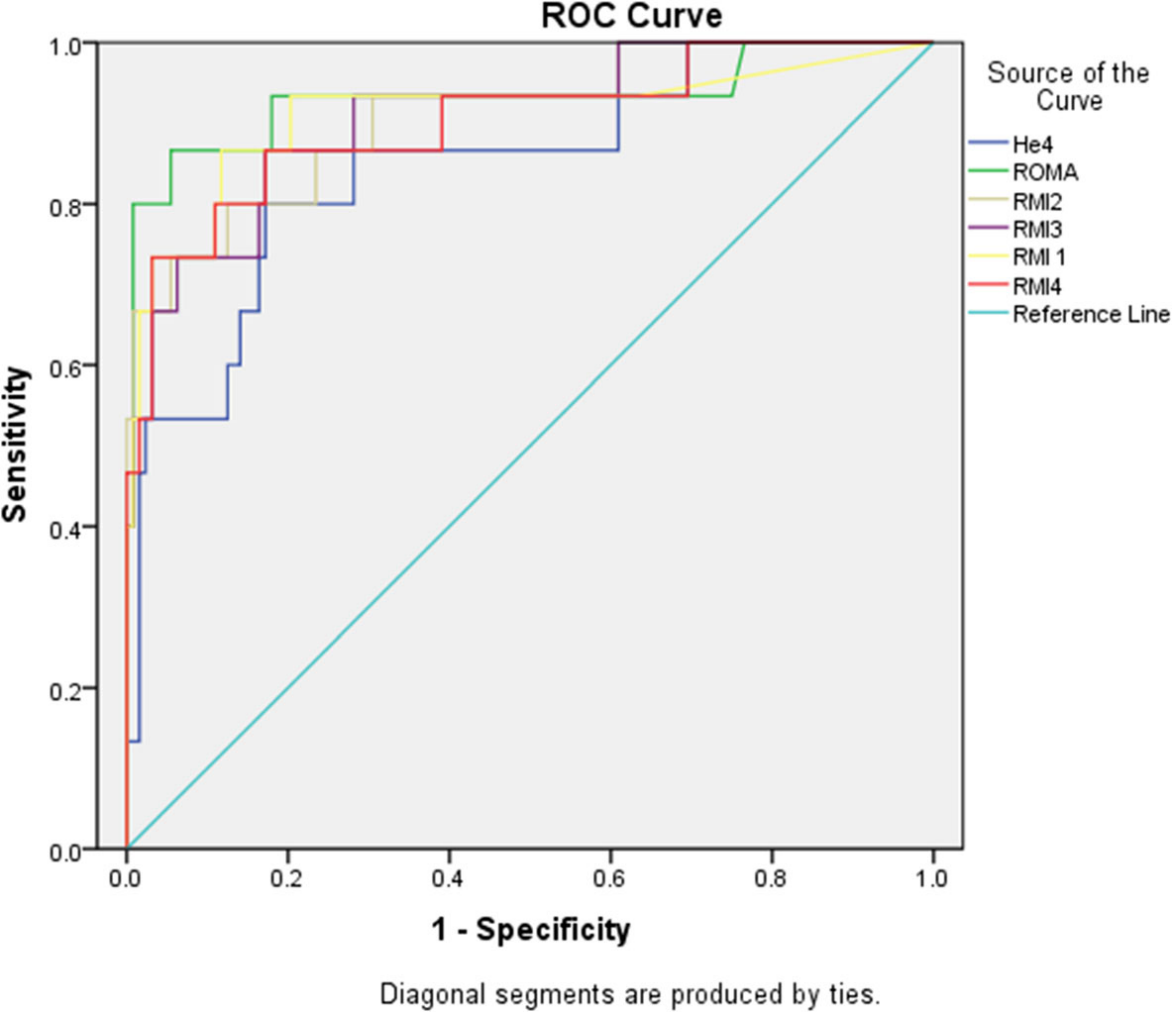
ROC curve benign vs epithelial ovarian cancers

The sensitivity, specificity, positive predictive value (PPV), negative predictive value (NPV) and Area Under the Curve of the six tests are depicted in Table 4. RMI2 and RMI 4 demonstrated the highest sensitivity whereas HE4 test demonstrated highest specificity. Comparison of the AUCs of the tests at 95% confidence interval showed no significant difference between the tests (Fig. 1). For epithelial tumors, ROMA demonstrated the highest sensitivity compared to other tests but the AUCs of all tests at 95% confidence intervals were comparable without significant differences as depicted in Table 5 (Fig. 2).

**Table 4:** Comparison of diagnostic performance of tests

Test	Sensitivity %	Specificity %	PPV%	NPV%	Area under the curve (95% confidence interval)
RMI1	63 (17/27)	93.8 (120/128)	68 (17/25)	92.3 (120/130)	.844 (0.740–0.947)
RMI2	66.7 (18/27)	89.1 (114/128)	56.3 (18/32)	92.7 (114/123)	.851 (0.762–0.940)
RMI3	63 (17/27)	90.6 (116/128)	58.6 (17/29)	92.1 (116/126)	.841 (0.749–0.932)
RMI4	66.7 (18/27)	92.2 (118/128)	64.3 (18/28)	92.9 (118/127)	.841 (0.744–0.938)
ROMA	59.3 (16/27)	93 (119/128)	64 (16/25)	91.5 (119/130)	.886 (0.805–0.967)
HE4	37 (10/27)	96.9 (124/128)	71.4 (10/14)	87.9 (124/141)	.798 (0.704–0.892)

Data represented as percentages at 95% confidence interval *PPV* positive predictive value, *NPV* negative predictive value, *RMI* Risk of malignancy index, *ROMA* Risk Of Malignancy Algorithm, *HE4* Human Epididymis Protein 4. Cut off for RMI1, RMI2, RMI3 was 200, for RMI4 was 450; for ROMA cut off for premenopausal was 11.4, for postmenopausal was 29.9; for HE4 cut off of 70 pmol/l was used

**Table 5:** Comparison of diagnostic performance of tests for epithelial ovarian cancers

Test	Sensitivity%	Specificity%	Area under the curve (95% confidence interval)
RMI1	73.3	93.8	0.861 (0.731–0.992)
RMI2	73.3	89.1	0.847 (0.715–0.979)
RMI3	73.3	90.6	0.861 (0.731–0.992)
RMI4	73.3	92.2	0.868 (0.742–0.994)
ROMA	80	93	0.861 (0.731–0.992)
HE4	53.3	96.9	0.653 (0.479–0.827)
Pre-menopausal			
RMI 1	42.9%	91.5%	0.672 (0.432–0.912)
RMI 2	42.9%	87.7%	0.653 (0.416–0.890)
RMI 3	42.9%	87.7%	0.653 (0.416–0.890)
RMI 4	42.9%	90.6%	0.667 (0.428–0.907)
HE4	42.9%	98.1%	0.705 (0.459–0.950)
ROMA	42.9%	91.5%	0.672 (0.432–0.912)
Menopausal			
RMI 1	72.2%	100%	0.861 (0.731–0.992)
RMI 2	77.8%	91.7%	0.847 (0.715–0.979)
RMI 3	72.2%	100%	0.861 (0.731–0.992)
RMI 4	77.8%	95.8%	0.868 (0.742–0.994)
HE4	77.8%	91.7%	0.653 (0.479–0.827)
ROMA	77.2%	100%	0.861 (0.731–0.992)

Data represented as percentages at 95% confidence interval

*PPV* positive predictive value, *NPV* negative predictive value, *RMI* Risk of malignancy index, *ROMA* Risk Of Malignancy Algorithm, *HE4* Human Epididymis Protein 4. Cut off for RMI1, RMI2, RMI3 was 200, for RMI4 was 450; for ROMA cut off for premenopausal was 11.4, for postmenopausal was 29.9; for HE4 cut off of 70 pmol/l was used

**Fig. 3:**
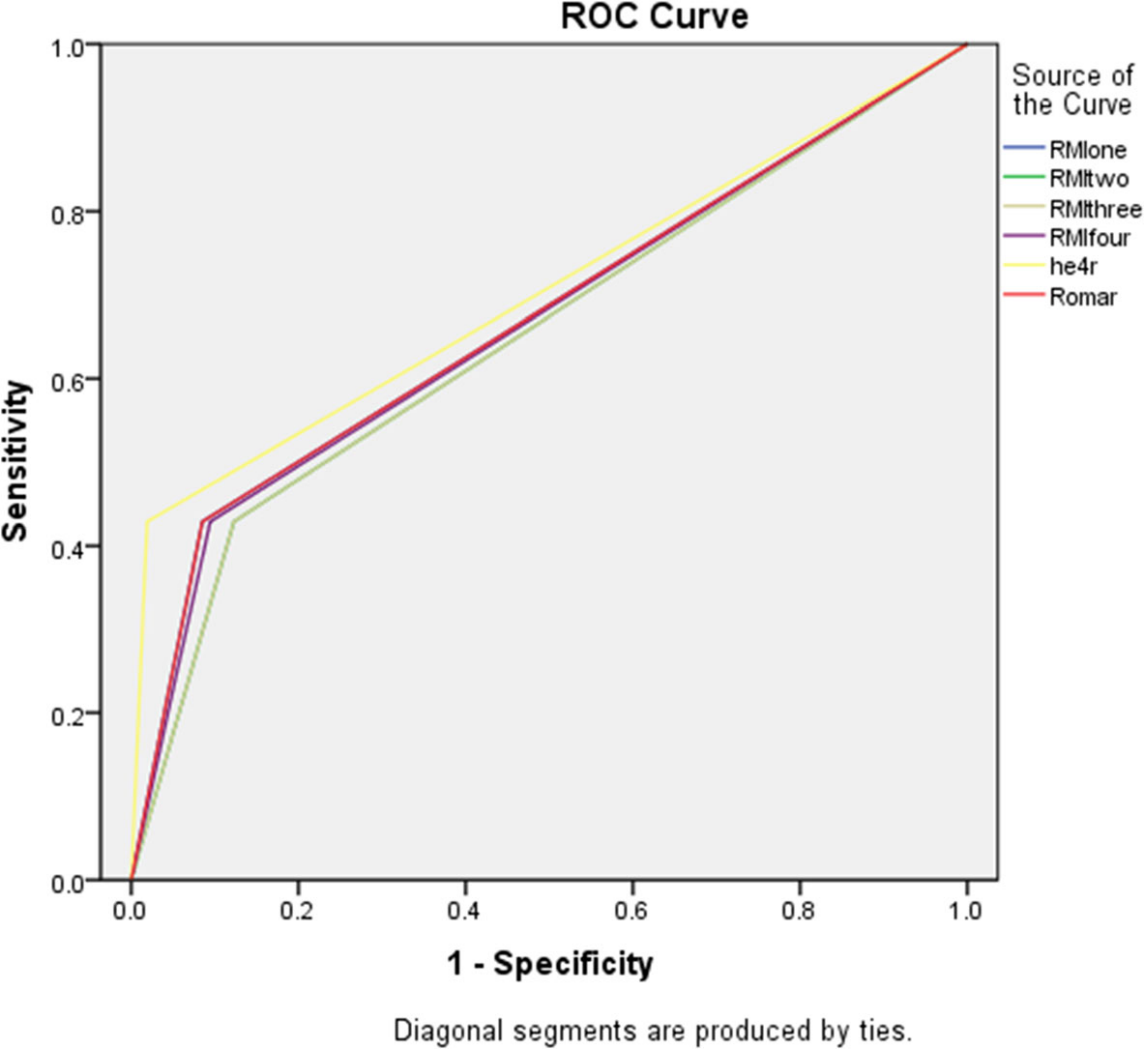
ROC curve benign vs all ovarian cancers in premenopausal

**Fig. 4:**
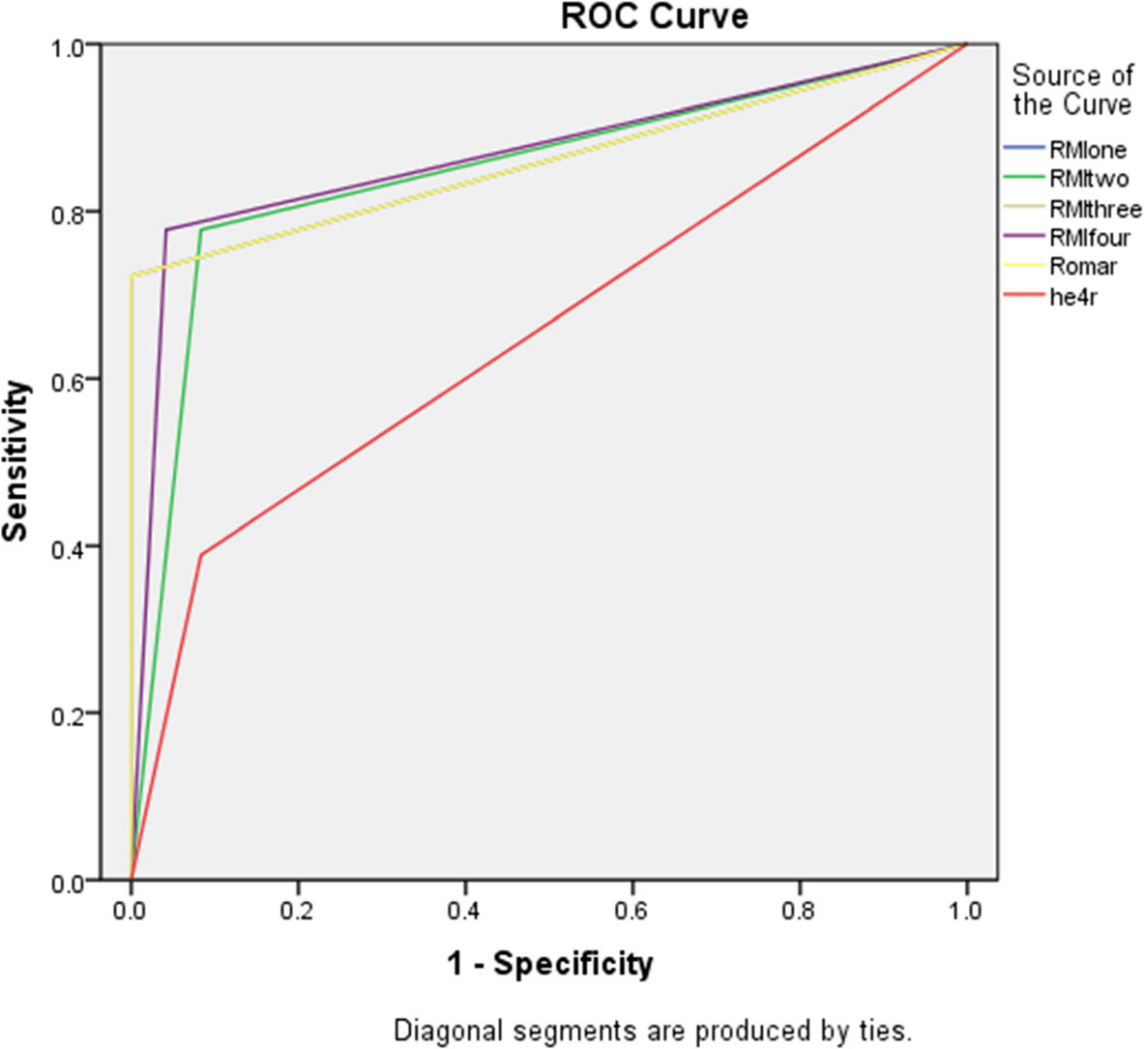
ROC curve benign vs all ovarian cancers for postmenopausal

The sensitivity of all the six tests were lower in the premenopausal group than those in the menopausal group. In the pre- menopausal group, the HE4 had the highest AUC of 0.705 but there was no significant difference when compared to the AUCs of other tests (Fig. 3). In the post-menopausal group as well, the AUCs of the tests at 95% confidence interval did not show significant differences (Table 6, Fig. 4).

**Table 6:** Comparison of performance of tests in premenopausal and postmenopausal groups

	Sensitivity	Specificity	Area Under the Curve (95% confidence interval)
Pre-menopausal			
RMI 1	42.9%	91.5%	0.672 (0.432–0.912)
RMI 2	42.9%	87.7%	0.653 (0.416–0.890)
RMI 3	42.9%	87.7%	0.653 (0.416–0.890)
RMI 4	42.9%	90.6%	0.667 (0.428–0.907)
HE4	42.9%	98.1%	0.705 (0.459–0.950)
ROMA	42.9%	91.5%	0.672 (0.432–0.912)
Menopausal			
RMI 1	72.2%	100%	0.861 (0.731–0.992)
RMI 2	77.8%	91.7%	0.847 (0.715–0.979)
RMI 3	72.2%	100%	0.861 (0.731–0.992)
RMI 4	77.8%	95.8%	0.868 (0.742–0.994)
HE4	77.8%	91.7%	0.653 (0.479–0.827)
ROMA	77.2%	100%	0.861 (0.731–0.992)

*RMI* Risk of malignancy index, *ROMA* Risk Of Malignancy Algorithm, *HE4* Human Epididymis Protein 4

## Discussion

Triage of adnexal masses presenting in the clinical setting between benign and malignant etiologies is crucial in order to guide the correct line of management. Benign masses can be managed locally whereas malignant masses are best treated in tertiary care centers by gynecologic oncologist. Ovarian cancer is often diagnosed at an advanced stage and carries a poor prognosis. Therefore characterization of the mass is important not only to determine whether a referral is needed but also to decide the line of management which has a significant impact on overall prognosis. Realizing the pressing need for the development of a system that can correctly differentiate the pelvic mass into benign or malignant, various efforts have been made and various scoring systems, marker analysis and prediction models have been developed. Despite these attempts there remains a lack of universally accepted testing methodology. This study aimed to compare the performance of RMI 1, RMI 2, RMI 3, RMI 4, HE4 and ROMA in discriminating benign and malignant pelvis masses.

Risk of Malignancy Index, first published by Jacobs et al. is a scoring system based on scores from ultrasound (U), menopausal status (M), and CA-125 data in the following manner: RMI=U × M × CA-125. The original cutoff used in the study was 200 and using the sample size of 143, the sensitivity and specificity obtained were 85.4 and 96.9 respectively [3]. According to the meta -analysis conducted by Kaijser et al., the pooled summary estimates for sensitivity and specificity from 23 studies were 72% (67–0.76% CI) and 92% (89–0.93% CI) respectively [10]. RMI 2 was proposed by Tingulstad et al. in 1996 modifying the scores assigned to the ultrasound and menopause components of RMI 1 using the cutoff level of 200. The original study include 173 patients and the sensitivity and specificity obtained were 71 and 96% respectively. The pooled summary estimates for sensitivity and specificity from 15 studies were found to be 75% (69–80% CI) and 87% (84–90% CI) [10].

In 1999, Tingulstad et al. further modified the two previous RMI models, and introduced RMI 3 assigning new scores to the ultrasound and menopause components using the same cut off level. The original study included 365 patients with obtained sensitivity and specificity of 71 and 92% respectively [5]. The pool summary of 9 studies conducted reflects the sensitivity of 70% (60–78% CI) and specificity of 91% (88–93% CI) [10]. In 2009, Yamamoto et al. developed the RMI 4 which included tumor size (S) as an additional component to previous versions of RMIs using a cutoff level of 450. In their retrospective study with 253 cases RMI 4 was found to have sensitivity and specificity of 86.8 and 91 respectively. The pool summary of 3 studies conducted reflects the sensitivity of 68% (59–76% CI) and specificity of 94% (91–96% CI) [10].

The serum biomarker HE4 was introduced as a novel and promising marker by Hellstrom et al. [11] and in 2008 has been cleared by the U.S. Food and Drug Administration for ovarian cancer monitoring. Moore et al. in 2009 introduced a biomarker based algorithm based the combination of two pilot studies, ROMA which combines the results of HE4, CA125 and menopausal status to calculate a risk score and categorizes the mass as high risk or low risk for malignancy. It was then subsequently validated in a multicenter trial assessing women presenting with pelvic mass. While evaluating 531 pelvic mass patients, the sensitivity for benign vs malignant was found to be 93.8% at specificity of 75%; 88.9% for premenopausal and 94.5% for post menopausal women at a fixed specificity of 75% [12, 13].

This study was conducted to compare the performance of each test for the same set of cases presenting with pelvic adnexal mass. In this study it was found that RMI 1, RMI2, RMI3, RMI4, HE4 and ROMA were all able to differentiate between benign and malignant masses. The borderline tumors when considered in the benign group, higher sensitivity were obtained for each tests. The sensitivity, specificity and AUC for the tests when overall tumors were considered were 63, 93.8% and 0.844 (RMI1); 66.7, 89.1% and 0.851 (RMI2); 63, 90.6% and 0.841 (RMI3); 66.7, 92.2% and 0.841 (RMI4); 59.3, 93% and 0.886 (ROMA) and 37,96.9% and 0.798 (HE4) respectively.. The difference in AUC between the tests were not found to be significant. When median values of the test for benign, borderline and malignant tumors were compared, no test was found to have significantly different value for benign and borderline tumors and all except for HE4 had significantly different median values for borderline and malignant tumors. When only epithelial ovarian cancers were considered, it was found that the sensitivity of each test was higher compared to that when overall tumors were considered: 73.3% (RMI 1), 73.3% (RMI2, RMI3, RMI4), 80% (ROMA) and 53.3% (HE4). The ROMA had the highest sensitivity in that case but the AUC between different tests were not found to be significantly different. The sensitivity of the tests were found to be lower in the premenopausal group than that in postmenopausal group.

## Conclusion

RMI 1–4, ROMA and HE4 were all found to be useful for differentiating benign/borderline adnexal masses from malignant ones for deciding optimal therapy, however no test was found to be significantly better than the other. None were able to differentiate between benign and borderline tumors. All of the tests demonstrated increased sensitivity when borderline tumors were considered low-risk, and when only epithelial ovarian cancers were considered.

## Data Availability

The datasets generated during and/or analysed during the current study are available from the corresponding author on reasonable request.

## Abbreviations

RMI: Risk of Malignancy Index
ROMA: Risk Of Malignancy Algorithm
HE4: Human Epididymis Protein 4
PPV: Positive Predictive Value
NPV: Negative Predictive Value
AUC: Area Under the Curve
ROC: Receiver Operator Characteristics
